# An IoE and Big Multimedia Data Approach for Urban Transport System Resilience Management in Smart Cities

**DOI:** 10.3390/s21020435

**Published:** 2021-01-09

**Authors:** Emanuele Bellini, Pierfrancesco Bellini, Daniele Cenni, Paolo Nesi, Gianni Pantaleo, Irene Paoli, Michela Paolucci

**Affiliations:** 1 Department of Mathematics and Physics, University of Campania, 81100 Caserta, Italy; 2 Distributed Systems and Internet Technology Lab DISIT, University of Florence, 50121 Firenze, Italy; pierfrancesco.bellini@unifi.it (P.B.); Daniele.Cenni@unifi.it (D.C.); paolo.nesi@unifi.it (P.N.); gianni.pantaleo@unifi.it (G.P.); irene.paoli@unifi.it (I.P.); michela.paolucci@unifi.it (M.P.)

**Keywords:** smart resilient city, big multimedia data, complex system, big data analysis, disaster resilience, evidence driven decision support system, functional resonance analysis method, Internet of Everything, tree value logic

## Abstract

Today, the complexity of urban systems combined with existing and emerging threats constrains administrations to consider smart technologies and related huge amounts of data generated as a means to take timely and informed decisions. The smart city needs to be prepared for both expected and unexpected situations, and the possibility to mitigate the effect of the uncertainty behind the causes of disruptions through the analysis of all the possible data generated by the city open new possibility for resilience operationalization. This article aims at introducing a new conceptualization for resilience and presenting an innovative full stack solution to exploit Internet of Everything (IoE) and big multimedia data in smart cities to manage resilience of urban transport systems (UTS), which is one of the most critical infrastructures of the city. The approach is based on a novel data driven approach to resilience engineering and functional resonance analysis method (FRAM), to understand and model an UTS in the context of smart cities and to support evidence driven decision making. The paper proposes an architecture taking into account: (a) different kinds of available data generated in the smart city, (b) big data collection and semantic aggregation and enrichment; (c) data sense-making process composed by analytics of different data sources like social media, communication networks, IoT, user behavior; (d) tools for knowledge driven decisions able to combine different information generated by analytics, experience, and structural information of the city into a comprehensive and evidence driven decision model. The solution has been applied in Florence metropolitan city in the context of RESOLUTE H2020 research project of the European Commission.

## 1. Introduction

According to the United Nations Population Fund by 2030, roughly 66 percent or 5 billion people, will live in urban areas. Therefore, the demand for services in urban areas is increasing exponentially in parallel with the city complexity.

Jointly, climatic extreme events and disasters may intensify or become more frequent in regions not accustomed to cope with such events [1]. Therefore, even if a city can reach a good understanding of the kind of threats and severity it is potentially exposed to, (by means conventional risk analysis [2] or recalling past experiences), the damage caused by extreme events occurred in recent years, reveals a dramatic lack of awareness and preparedness impacting on the capacity of a community to absorb, quickly recover, and learn from the disruption. For instance, according to the JRC research outcomes [3], the number of deaths from weather disasters could increase 50-fold in Europe by the start of the next century, if no measures for adaptation are taken. Assessing the impact on Europeans over a 30-year interval, period 2071–2100, JRC found that two out of three people in Europe might be affected by weather-related disasters annually, which makes an estimate of 351 million people. By contrast, between 1981 and 2010, 25 million people had been exposed: just 5% of Europe’s entire population. However, the analysis assumed no reduction in human vulnerability or city resilience enhancement over time thanks to adaptation.

The need to better understand the causes of disasters through the analysis of all the possible data generated by the city to be prepared for an expected and unexpected situation is evident [4]. The global trend of smart city implementation has brought significant investments to deploy every kind of smart technologies (e.g., environmental sensors, traffic sensors, public Wi-Fi) in the urban area with the aim of increasing awareness and control on city events and dynamics. Moreover, the level of “smartness” [5] and data generated are increased in many cities around the world making a number of events detectable (e.g., river level, pollution, wind speed, traffic flows, human movements), opening new possibilities to manage urban resilience through a data driven approach. According to [6], the boost to efficiency and optimization that underline smart city implementation fails to acknowledge secondary effects and feedbacks that cause changes in the system as a whole. Overall, the increased complexity and fast pace changing nature of urban systems are generating many emergent challenges, in the face of which most currently used management and operational practices have demonstrated many shortfalls as explained in [7]. In particular:

(a) the underspecified nature of operations in complex systems (many adverse events are the result of unexpected combinations of normal performance variabilities [8]) generates potential for unforeseeable failures and cascading effects;
(b) the existence of multiple sub-systems with non-linear and sometimes hidden interactions, requires approaches to cope with “unknown unknowns” that is not always fully understood;
(c) existence of a number of methods and scales of analysis not fully standardized;
(d) multiple stakeholders and institutions which have different worldviews and competing opportunistic goals.

Facing such issues requires the adoption of a holistic view able to guide ICT investments and deployments toward shared and well-defined goals, namely city resilience and sustainability, is needed. Cities need to strengthen the science-policy interaction, to properly assess their metabolism dynamics, and to increase socio-ecological sustainability and resilience in facing known and unknown challenges.

In order to cope with above mentioned issues, this article aims to investigate the possibility of adopting a big data driven approach for resilience management in a socio-technical system as a smart city.

The paper is focused on a new conceptualization for resilience suitable for the purposes and on a full-stack solution developed in the framework of the RESOLUTE European Commission research and development project (http://www.resolute-eu.org). The approach exploits the opportunity given by the Internet of Everything paradigm, to collect, process and transform the urban big multimedia data (U-BMD) generated within the system by people and “things”, into valuable knowledge for decision making in resilience and sustainability. The focus is on the urban transport system considered as the set of transport infrastructures and modes that support urban movements of passengers and freight. Today urban transport systems (UTS) have developed a prominent safety and business critical nature, thus enhancing resilience of UTS is considered imperative for two main reasons: (i) it provides essential support to every socio-economic activity and rescue; (ii) the paths that convey people, goods, service and information, are the same through which risks are propagated [9]. In fact, the increment of the population in urban areas, the increasing interdependencies among physical and cyber infrastructure, the neighboring of transportation systems with hazardous production facilities—along with the threats of climate change and terrorism—are creating significant challenges for the UTS as a critical infrastructure system. The present paper aims to provide methods and technologies to operationalize UTS resilience exploiting the data generated in the smart city.

The article is organized as follows: in Section 2 is presented the related work; in Section 3 is defined the methodology to exploit U-BMD; in Section 4 is introduced the 3-TierS Resolute U-BMD Architecture to operationalize resilience management; in Section 5 is described the application of the RESOLUTE Architecture to a smart city scenario based on different data sources and data analysis; in Section 6 are provided the conclusions and forthcoming steps.

## 2. Related Work

### 2.1. Resilience

Resilience is a multi-faced and not yet standardized concept so that a number of definitions and assessment methods exist [10]. Resilience has emerged as an attractive concept with respect to cities when they are started to be considered as complex and adaptive socio-technical systems [11]. The emerging notion of “resilience thinking” inspired by that of “system thinking”, offers a new way of understanding such system complexity and a new approach to manage their resources to continually adapt through cycles of change in order to achieve sustainability [6].

The definitions and conceptualizations of resilience present in literature such as [12,13,14,15] can be roughly clustered into two views: (1) resilience as a performative property derived by the system ability to cope with changing conditions; and (2) resilience as an emerging property derived by the system adaptive capacity.

**Figure 1:**
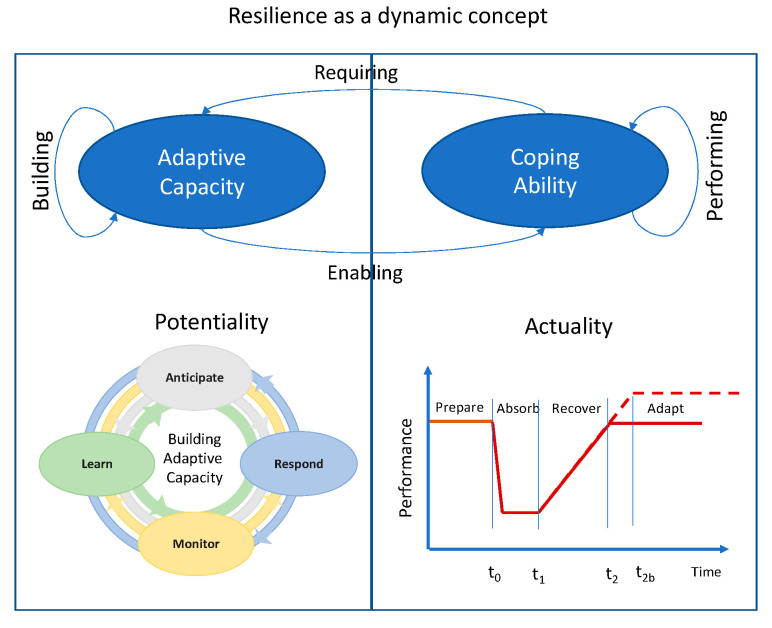
Resilience as a dynamic concept. The adaptive capacity is built through a continuous process where the four resilience cornerstones are implemented. The coping ability is exhibited as soon as an event occur and follows the four phases: prepare, absorb, recover, and adapt.

The first one is based on the National Academy of Science definition [13] that adopts a “functionality-based” [14] view. In particular, resilience is exhibited along four temporal based phases (plan, absorb, recover, adapt) during which the functionality of a system is monitored and the level of resilience is quantified within a specific time window. In other words, the functionality Q(t) is measured as a percentage function of time, and the resilience is quantified as the integral of the functionality function (see Figure 1 Actuality part) [15]. In this view, the resilience can be assessed only though numerical approach (simulation) or after the conclusion of the event. The performance-based perspective of resilience is also adopted by the 100 Resilient Cities (100RC) initiative—supported by The Rockefeller Foundation. In fact, while resilience engineering focuses the actual capability of a system of adjusting its functioning on changing conditions, the 100RC perspective seems to be looking at the performance (the actuality) obtained during acute shocks as well as chronic stresses [16].

The second view is based on concept adaptive capacity and seems to be more suitable to treat city resilience. In fact, more than half of the urban resilience definitions retrieved in the review carried out in [17], associate generic adaptability, flexibility, or adaptive capacity concepts to urban resilience. The concept of the adaptive cycle for cities is derived by the panarchy theory [18] grounded on ecosystems dynamics. As defined in [19], the panarchy related to a city includes the definition of a dynamic set of urban adaptive cycles that evolve, irreversibly and uniquely, as they adapt to new stressors (e.g., resource demands, crisis). A city—as a complex socio-technical system—should be considered stochastic, dynamic, and unpredictable by nature. In this respect, urban resilience should be related with the system performance, and its capability of controlling and coping with continuous changing conditions. In particular, sustaining this adaptability requires overall enhanced operational efficiency, mainly by optimizing the allocation and utilization of available resources whilst striving to continuously minimize operational failures. Within this context, resilience can be seen as an emergent property of a complex system and it is about managing high variability and uncertainty in order to continuously pursue successful performance of a system.

In this perspective, as argued in [12], only the “potential” for resilience can be actually observed and quantified in a system, and not the resilience itself. Such an intrinsic capability of a system to adjust its functioning prior to, during, or after an expected or unexpected stressing event, is oriented to continually sustain system operations [20]. Thus, this “potential” for resilience can be evaluated against the so called “resilience cornerstones” [21,22], namely:

- Respond (knowing what to do): it is related to the capacity of the system, to respond to a stressor by continuously adjusting system performance to changing conditions.
- Monitor (knowing what to look for): it is related to the capacity of the system, to monitor both the system and the context collecting data and information to detect events and reduce the uncertainty.
- Anticipate (knowing what to expect): it is related to the capacity of the system to early identify and evaluate potential threats as well as their consequences for system operation seizing the opportunities for changes offered by the needs of adaptation.
- Learn (knowing what has happened): it is related to the capacity to learn from past experiences either successful or not.

In summary, the essence of resilience lies in the system capacity to recognize when variability in its performance is unanticipated and falls beyond the expected range and to actively dampen such variability through continuous adaptation [14]. In order to cope with such a variability, the limited capacities of the system should be known and managed to achieve the right synchronization and coordination level [2].

### 2.2. Smart City and Internet of Everything

The new global trend that moves toward the Internet of Everything (IoE) implementation contributes to accelerate the evolution of the city that become even more smart. The IoE represents an enabling paradigm to enhance the resilience in smart city, contributing to monitoring, detecting, and dampening the unwanted and unexpected variability caused by a critical event [7]. The IoE can be considered a natural evolution of the Internet of Thing (IoT) concept [23,24,25], In fact, the IoE includes the following four key elements [26,27,28]:

(a) People: considered as end-nodes always connected to the Internet and as a source of knowledge, information, decisions, behaviors and so forth.
(b) Things: it ranges from physical sensors and actuators with limited computational capabilities, to smart devices (e.g., smartphones) able to generate and process a relevant amount of data as multimedia resources.
(c) Big Multimedia Data: huge stream of raw data generated, exchanged analyzed and processed to enable reliable decisions and control mechanisms,
(d) Processes: methodologies and mechanisms for automation to leverage high speed connectivity represented by the 5G (and the future 6G) among data, things, and people to add value.

The IoE paradigm helps to manage an end-to-end ecosystem of connectivity cantered on people and on their relationships, social collaborations, and grouping dynamics (connected community) within the smart city, enabling a proactive participation of the citizens in building the city resilience [2,27]. The collaboration of the people that act “as a (intelligent) sensor” implies the exploitation of the social media as a source of complex signals that needs to be collected, filtered, elaborated, and interpreted. In fact, as highlighted in [29] and [30], data from social media can be inaccurate, extemporaneous, malicious, and/or noisy. To this end, the goal of reducing uncertainty and making reliable decisions for the resilience of the city cannot be achieved by relying on this data source only and a clever integration of different sources is required.

According to [31], the data collection process and management to provide the aid, relief, and response in critical situations, have always challenged public decision makers, emergency services and disaster management community due to: (1) time constraints; (2) scattered nature of data sources, owners, and types; (3) speed of access to data; and (4) huge data volume. In fact, a smart city generates different multimedia data types that can be classified into the following two macro categories:

Batch/periodic: it includes points of interests, geo-referenced services, GIS based maps (e.g., flooding susceptibility map), accidents statistic, satellite/LiDAR images, etc. This information is typically accessible in several diverse both open and proprietary formats, such as: SHP, TIFF, KML, CVS, ZIP, XML, XLS, etc.

Near real time/real time: it includes data coming from IoT/wireless sensor networks of urban sensors (e.g., free spaces in parking, air temperature, pollution measures, triage status, Wi-Fi, public transport system, smart lighting, etc.), wearable sensors, social media (e.g., Twitter, Facebook, Instagram), instant messaging applications (e.g., Telegram), volunteered geographic information (VGI) including those from navigators, volunteered reporting-based applications [32], and so forth.

**Figure 2:**
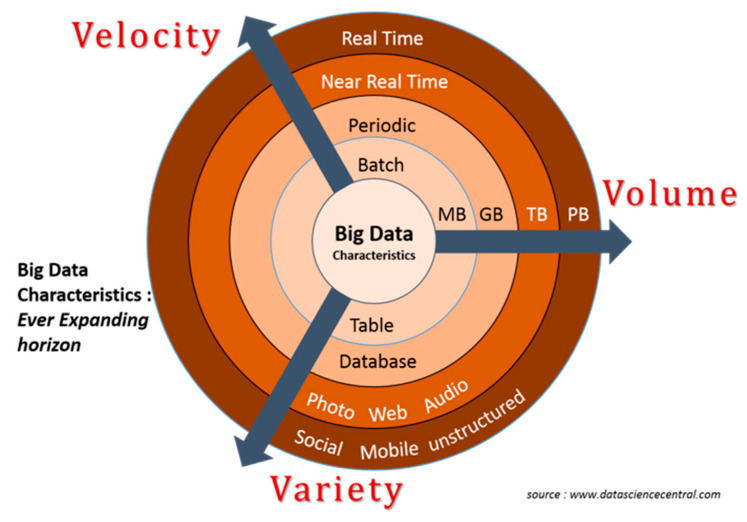
3V of big data (source: www.datasciencecentral.com).

Until now, resilience management has largely focused on descriptive (i.e., what happened?) or diagnostic analytics (i.e., why it did happen?) following an expert judgment-based approach [33]. Today, emerging technological innovations such as IoE (including social media, location-based systems, and big data analytics—BDA) have opened up the opportunity for real time monitoring (i.e., what it is happening?), thus, a dynamic evidence driven system resilience management [2,14,34], and predictive analytics based on increasingly reliable big data (i.e., what it will happen?) is now at hand. Ad depicted in Figure 2, Big data are usually related to the 3V (velocity, volume, variety), but the perspective is becoming wider with 4V (+value), 5V (+veracity) [35], etc. According to [36], a fourth V has been added to the standard 3V, namely ‘value’ and identified as the capability of the big data, to generate a public value in different contexts, such as: business, social, political, etc.

**Figure 3:**
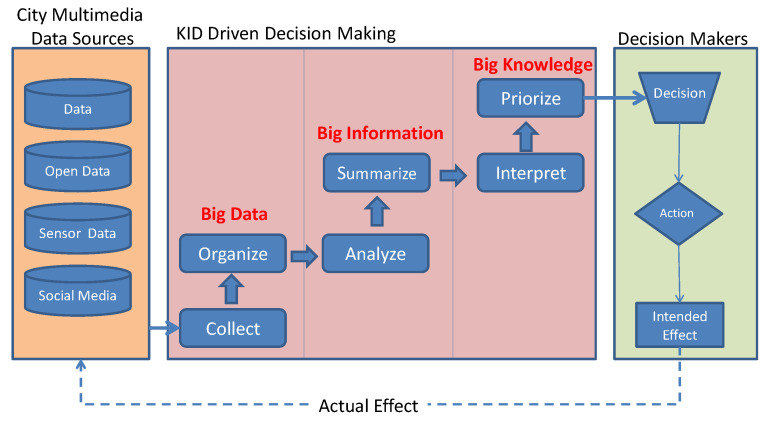
KID (knowledge, information, data) driven decision-making process in urban context. Different data sources available in the smart city should be properly processed to became valuable knowledge for decision makers.

The availability of such a quantity of information in smart city actually leads to a question: what about their actual usefulness? There are valuable examples of analysis based on big data and IoT as reviews in [37]. However, is possible to turn its cost into value for resilience-based decisions? To this end, it is necessary to transform U-BMD from raw state into information and then into knowledge pandering the sort of continuum that such three elements as stated in [38]. In fact, if a single data source is taken alone, it does not carry any implication or useful deduction for future actions. This can be done effectively only transforming smart city data into knowledge. Knowledge is created through a process of information interpretation, contextualization, and prioritization, whose results are used to guide decisions. Figure 3 depicts such a transformation process which has been adopted in present work and adapted from [36].

Every decision aims at generating an effect. The effect of such a decision becomes evident by means of new U-BMD generated by the actors affected by these decisions. Such control loop grants a continuous adjustment of the decisions during the disaster emergency, to both: (i) align the effects to the intentions and (ii) reduce time needed for recovery and bounce back (or forward) to desired conditions. In order to translate U-BMD into value for the disaster resilience management, the following steps must be taken: (a) understanding the system; (b) understanding informational needs for decision making; (c) selecting/producing data needed to support such decisions; (d) transforming data into knowledge; (e) supporting decision making.

## 3. Method

### 3.1. Towards a Novel Definition of Resilience

However, a clear integration of the two concepts is still lacking at theoretical and operational level in literature. To this end, in the present work, a reconciliation has been proposed decomposing resilience concept in two different foundational but inherently interlinked elements: adaptive capacity and the coping ability. Resilience emerges as a result of a dynamic process between them through which successful performance is continually pursued [39]. According to this view, the adaptive capacity represents the potentiality of the system that needs to be continuously built along the four cornerstones (anticipate, respond, monitor, learn) in order to enable the full expression of the system coping ability in terms of survivability, adaptability, and sustainability.

Coping ability represents the exploitation of the resilience potential (adaptive capacity) and it is exhibited during a specific critical event. This means that it can be evaluated in terms of system functionality dynamics (loss and recovery) along the time but only in relation to a specific unexpected event. The definition of what is a triggering event is difficult in resilience management. In risk analysis the events to be considered are pre-defined and the uncertainty remains mostly related to the effects side (magnitude, position, etc.). On the other hand, when unknown events occur and high level of uncertainty is also related to the causes of the event (unknown unknowns), the coping ability is expressed by how performance levels are maintained over time through adaptation to changing conditions. Such a continuous adaptation performance is difficult to be assessed in socio-technical systems because of the underspecified nature of operations (e.g., the human performance in an emergency is not fully predictable only on the base of the skill owned) [8]. According to this, in the present work we show a significant enhancement in the UTS adaptive capacity in relation to the introduction of smart technologies such as U-BMD.

### 3.2. Functional Resonance Analysis Method

The functional resonance analysis method (FRAM) [40] is a modeling tool for complex systems that focuses on system functions, their interdependencies, and dynamics.

A FRAM function that could have a human, technological or organizational nature, contributes to transforms the state of the system towards fulfilling the operational purpose. This introduces in the modeling a diversity of factors relating to system dynamics, which frequently are unobserved within models based on organizational structures or process flows. FRAM takes into account the non-linear nature of performance in complex systems, as opposed to building cause-effect sequences of events in time. The accidents are produced by unexpected interaction and composition of function performance variabilities. Hence FRAM supports resilience assessment by providing an understanding mechanism towards damping the sources of variability. In this respect, FRAM is based on four basic principles:

(a) The equivalence of success and failure that emerge from performance variability.
(b) Variability represents the deviation of the performance respect to the expectation.
(c) Emergence of either success or failure is due by the unexpected interaction of variability of different functions.
(d) The unexpected ‘amplified’ effects of interactions between different sources of variability are at the origin of the so-called functional resonance phenomena that leads to a disruption.

**Figure 4:**
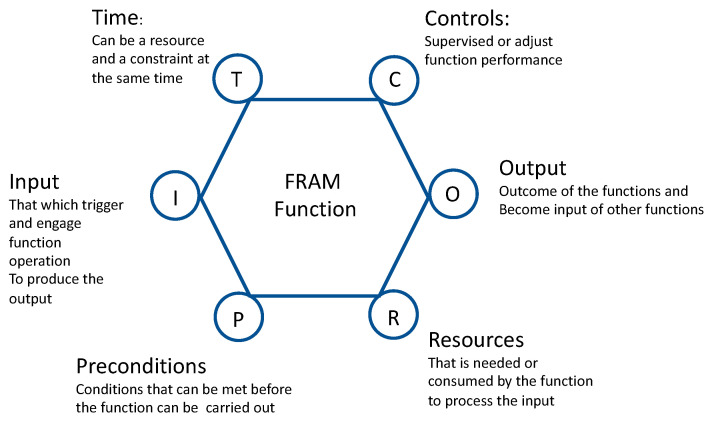
Functional unit of FRAM.

The fundamental step in the use of this method is the identification and description of functions and their interdependencies. In Figure 4, the FRAM function is represented. Each function is defined by six descriptors (time, control, output, resource, precondition, and input). A function refers to the activity—or to a set of activities—of the system which are required to produce an expected outcome.

The analysis of the potential sources of variability represents the entry point of the assessment of the system capacities needed to cope with expected and unexpected variability emerging from system operation. The method is designed to solve the gap existing between the understanding of how the system is imagined to work and how it actually works [39]. The method is at the base of retrospect analysis but it is also promising for the prospective one, and in particular for the real time quantitative approach [41].

Several initiatives are exploring the possibility to extend FRAM towards a quantitative analysis. In [42], has been exploited the expert knowledge using questionnaire and analytical hierarchy process to transform linguistic scale into numerical scale. The use of expert knowledge has been adopted also in [2], where the fuzzy logic has been used to translate judgments into values through membership functions. A similar approach has been followed also in [43].

A semi-quantitative analysis based on Monte Carlo simulation is proposed in [44]. The idea is to evaluate the variability of the output of a function quantifying the resulting effect (amplifying, neutral, dampening) of the coupling between upstream and downstream functions. These approaches represent the remarkable effort of the FRAM community to move towards quantitative analysis. However, these approaches to FRAM quantification are not based on the heterogeneous and dynamic data as generated by a smart city. Their utility remains confined at the analysis level while the intent of the present work is to transform the FRAM model into a tool to support close to real time data driven decision making in an operational setting.

In this respect, the introduction of a new point of view defined as “Work-As-Desired” [22] to build a reference model (instead of a representation or simulation of the reality) as a baseline (e.g., thresholds) for the assessment, represents one of the main contributions towards the integration of the data driven quantitative analysis in FRAM.

In particular, the recommended breadth-before-depth approach [45] has been followed and two subsequent steps has been defined:

First step: The definition of the desired functions that a system should comprise to be resilient (see Table 1).

Second step: Definition of the interdependencies (second step) that should be present to secure system resilience.

The obtained result is a reference model to be used to define indicators for each function of the systems. This allows the calculation of the system resilience index, a synthetic proxy indicator for resilience, able to quantify the system adaptive capacity [21,22,25,39]. It is worth noticing that the definition of the indicators should follow a consensus-driven process involving all the actors and stakeholders that in one way or another are connected to these indicators, both, because they can be the evaluators of these indicators or the evaluated [34,46]. Thus, the quantification of the enhancements of the adaptive capacity obtained with the introduction of ICT in smart city aims at representing valuable information for decision-makers at strategic, tactical, and operational levels.

### 3.3. Urban Big Multimedia Data Approach

In order to translate U-BMD into valuable knowledge and wisdom [38,47] for the resilience management, the following multi-steps approach has been defined and described in detail in the underlying sections:

1. Understanding the urban transport system (UTS): use of the FRAM approach in managing critical events
2. Understanding what information is needed to take decisions
3. Selecting/producing U-BDM: methodologies to be adopted to select and collect the data needed
4. U-BDM collection and integration: data collection
5. U-BDM sense making, how the data is transformed into information
6. Knowledge driven decision: how the information is transformed into knowledge

#### 3.3.1. Understanding the UTS System

According to [48], a socio-technical system can be analyzed basing on the functions it performs rather than by how it is structured. From this point of view, in order to evaluate the system performance variability, it is necessary to: (i) understand how likely each function of the system can vary; (ii) identify the interdependencies among the different functions. In order to obtain this result, the functional resonance analysis method (FRAM) [47] has been exploited. FRAM aims to capture the dynamics of complex systems considered as a set of coupled or mutually dependent functions, by modeling the non-linear dependencies and variability of each function. As explained by Hollnagel in [49], performance variability, that is, the range of results in a function or an overall system’s performance, highly depends on the variability of the working conditions of the system. Understanding, quantifying, and managing such a variability may reduce the impact of a critical event and speed up the recovery process supporting informed decision making [46]. Applying the FRAM approach in the UTS (urban transport system) and basing on the European Resilience Management Guidelines (ERMG), released by RESOLUTE project (http://www.resolute-eu.org), [39]), the UTS reference model can be defined by 18 functions (see Table 1). The functions and the related interdependencies have been defined following the “Work-As-Desired” [22] approach as introduced in Section 2. In particular, a several experts have identified the main desirable functions against which it is possible to assess a complex system, as UTS.

The six functions highlighted in green in the Table 1 are those in which the adoption of IoE and U-BMD may have a relevant impact in reducing the potential variability and uncertainty in the system (smart city). Therefore, the U-BDM to be managed must be oriented to support them.

#### 3.3.2. Understanding Information Needs

In order to understand decision makers’ needs in terms of information availability, it is necessary to engage city operators (fire brigades, civil protection, mobility operators, etc.) in the system requirements elicitation. Therefore, some focus groups and workshops, centered on critical scenarios (flash flooding, big floods, traffic jam, car accidents, bomb attacks, etc.) must be organized. The aim is to elicit both explicit and implicit operator’s knowledge on how the system works, when comparing such “as-imagined” performance against the actual performance carried out during the emergency (“work-as-done”). Such an analysis of results may identify several drawbacks (e.g., fragmented responsibilities, lack of data sharing and communication among operators) [9], which could be mitigated if some of the decisions taken by operators during disaster, are improved in terms of efficiency (timing, allocated resource, etc.) and effectiveness. A list of critical decisions to be enhanced as emerged during a number of RESOLUTE workshops and focus groups. In Table 2, the result of the mapping between the critical decisions and the UTS functions most involved, is provided.

Most of these decisions require a comprehensive knowledge in order to be reliable in producing a positive effect at systemic level.

#### 3.3.3. Selecting/Producing U-BMD

Instead of collecting everything “just in case” or starting with the data you need to get access to, the approach suggested was to start by taking into account the system’s aims and interdependencies identified in the first step (understanding the UTS system) [50]. In this step the relevant data to be accessed or acquired is identified. It is important to remark that no data is inherently better or more valuable than another, until it is put in relation with other datasets and with the general decision-making criteria. This is subjected to a continuous process of improvement. In fact, every time a decision is taken, the analysts can realize that the level of uncertainty behind that decisions could be mitigated/reduced by using a different measurement or a dataset that is not actually available. This consideration has been used to drive the improvement of the monitoring capability generating new datasets/data streams as the Wi-Fi access data streams in real time.

#### 3.3.4. U-BMD Collection and Integration

Addressing the complexity of U-BMD collection is crucial. The kinds of data are very diverse in terms of volume, velocity, and variety, as well as in terms of accessibility and license for reuse. For instance, data generated within public utilities (energy, water, gas, etc.) like the GIS based pipeline positions or the operations data may fall within information security policies. Several kinds of U-BMD are available in a city: GIS maps (including seismic risk maps, hydrological risk maps, services, descriptors of structures such as schools, hospitals, infrastructures, etc.), social media streams, urban IoT data streams, video surveillance streams, etc. Such diversity must be managed through a flexible, scalable and comprehensive methodology and tools. In particular, the adoption of an ontology to semantically aggregate all the U-BMDs, can offer support to the subsequent contextualization and sense-making process.

#### 3.3.5. U-BMD Sense-Making

The U-BMD must be processed in order to transform data into information. All the collected data are tracks of events occurring in the city. The challenge is represented by the necessity of analyzing such tracks to extract significant facts (the “real signal”). For instance, the public Wi-Fi access data stream in a smart city, if properly processed (e.g., clustering analysis), may reveal concentration and trajectories of people in specific areas. Similarly, the analysis of the social media streams (e.g., Twitter) with natural language processing (NLP) and descriptive statistical techniques, may disclose the mood of the citizens, their opinions, some specific trends, etc.

#### 3.3.6. KID Driven Decisions

This step consists in the transformation of U-BMD derived information into knowledge. This transformation is enabled by the adoption of decision support systems (DSS). A DSS is a computer-based information system supporting organizational decision making [36,51,52]. The objective of a DSS is to provide support to decision makers to give them help to solve problems by gathering: (i) the decision makers’ expertise; and (ii) the data coming from the system; while analyzing them in a more intelligent and faster way with respect to humans [2].

## 4. U-BMD Architecture

### 4.1. Tier Architecture

**Figure 5:**
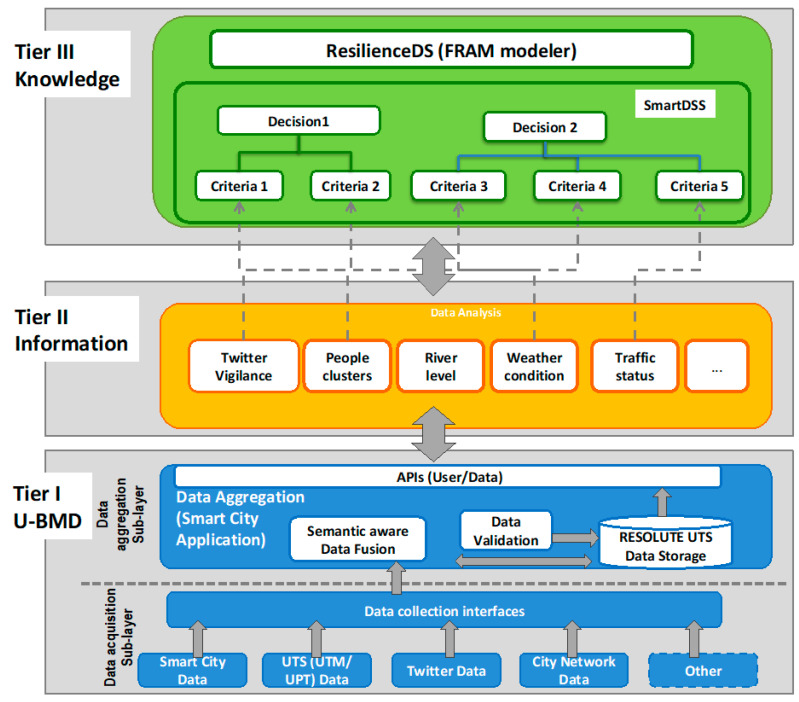
Three-tier RESOLUTE U-BMD Architecture. The architecture recalls the KID steps of data transformation for decision making presented in Figure 3.

In order to operationalize the approach, the RESOLUTE three-tier cloud-based architecture has been designed and implemented (see Figure 5). The tiers do not necessarily correspond to physical locations on various computers within a network, and rather they are the logical layers of the system functionalities. The three-tier architecture reflects the necessity to establish a set of technical procedures and approaches to produce new knowledge, useful to take decisions, starting from a large set of no correlated data and passing thought the information concept, described in Section 3. The tiers on which the architecture is based are:

- Tier I—Urban Big Multimedia Data Management
- Tier II—Information (U-BMD Sense Making)
- Tier III—Knowledge (Knowledge Driven Decision Support System)

The proposed approach can be classified at the 3rd tier according to the resilience assessment method classification defined in [53,54]. In fact, they not only can be considered a means to enhance the system resilience thanks to their capability of dampening the system variability, but they allow quantitative resilience assessment and data-driven resilience management. In particular, they enhance the for adaptive capacities as follows:

(a) Anticipate: by continuously assessing city vulnerabilities and identifying when the system operates closer to safety boundaries, predicting behaviors and event dynamics, supporting evidence-based decisions at strategic, tactical, and operational level, thus moving a step forward with respect to current practices based on pre-simulated emergency scenarios.
(b) Respond: by delivering real-time, context-aware, personalized, and ubiquitous advice to the citizens by exploiting IoE technologies
(c) Monitor: by improving the granularity, timelines, precision, quality and comprehensiveness of information about the city metabolism dynamics.
(d) Learn: by applying advanced analysis on U-BMD (e.g., deep learning, data analysis and prediction, sentiment analysis) to extract valuable information and knowledge for decision-making;

### 4.2. Tier I—Urban Big Multimedia Data Management

The urban big multimedia data management layer consists of two different sub-layers: data acquisition and data aggregation.

#### 4.2.1. Data Acquisition Sub-Layer

It implements the multi-source big data ingestion mechanism addressing aspects such as: variability, velocity, complexity, variety, geo-spatial aspects, integration, and size. In particular, a complex knowledge base is composed of two elements: (a) an RDF triple store based on an ontology which describes all the city elements (streets, services, buildings, sensors, etc.); and (b) a scalable NoSQL (HBase) to accommodate real time data generated by such elements [55].

It is well known that different types of datasets/data streams require different methodologies and approaches for their collection and ingestion. For instance, the ingestion of data relating to real time traffic sensors is implemented through an ETL (extract, transform, and load) process (e.g., Penthao Kettle) invoking a web service via HTTP Post managed by the owner of the data. In most cases, the real-time data are pushed directly into the mapping process to feed a temporary SQL store. They are typically streamed both into a traditional SQL store and then converted into triples in the RDF store with the Karma tool and in the NoSQL database (HBase). In some cases, real time data are streamed into an IOT broker first and then are into some NoSQL database and even driven IOT Applications.

The different kind of data can be grouped in the following main categories, also visible in Figure 4: Smart City Data, UTS Data, Twitter Data, City Network Data.

Smart City Data: it consists of a collection of static or slow dynamic open and private data coming from the city and its territory. Usually, this information is published by public and private organizations (e.g., city council, civil protection) in different file formats such as html, xml, csv, shp, etc. Many times, such kind of information are extracted from legacy systems and are the result of specific processing such as: number of visitors in a museum in a specific moment, weather forecasts for a specific area, events in the city, flooding vulnerability/susceptibility, and so forth.

UTS Data: it is related to datasets regarding mobility and transport aspects typically involved in a smart city [56,57], such as:

- Traffic Manager to track the status of the traffic in the city
- Public Transport schedule plans and real time status;
- Road network status: roads, bridges, underpasses, etc.;
- Parking position and status, car and bike sharing, movements of public vehicles, cycling paths, etc.

Social Media Data: it consists in collecting and analyzing data streams coming from Twitter by creating dedicated thematic channels, which can be tuned to monitor one or more search queries on Twitter with a sophisticated and expressive syntax. The solution has been set up only on Twitter data since it is a microblog platform strongly oriented to redistribution of post on real time.

City Network Data: it consists of data generated by mobile devices and/or networks acting in the city. In a smart city context, this type of channel is essential for taking care of the people/citizens’ presence and flow. An interesting example is represented by mobile device data collected by mobile phone operators, which can be used to determine the number of people connected/present in a city area. Unfortunately, the level of granularity (number of users in a certain instant) provided by the cells is about 1000 m per side, and therefore too large to extract detailed information of the citizens’ movement to be used for emergency management applications. Fundamental for analyzing the habits and activities of citizens is the information that can be extracted and properly anonymized from city Wi-Fi or IoT networks that offer a much higher degree of resolution. For example, they can be of great help in determining which services citizens use the most analyzing the dynamic of the concentration of the connections to a specific access point in the city. However, it should be remembered that the Wi-Fi data provides results only in relation to the location of the access points (AP). Therefore, if the city is not systematically covered by AP, we will have jagged information. One way to overcome this limitation is to use crowdsourcing through dedicated mobile apps that can be used to track user behavior and to perform measurements on the city, aiming to calculate origin–destination matrices, typical trajectories and warm places. For example, the “*Firenze dove cosa.*” (*Florence where what…*) App (available for both iOS and Android) allows this data collection by providing useful information for free. Even in this case, however, the monitoring is carried out only on the basis of the behavior of the population that installed the “City App”. Therefore, any assessment based on this information must take this limitation into account.

#### 4.2.2. Data Aggregation Sub-Layer

The data coming from the many different sources above described are semantically aggregated and are compliant with the Km4City Multi-Ontology [58]. The semantic relation among the different data plays a relevant role in supporting the U-BMD transformation into knowledge. Organizing the data according to a specific holistic view, formalized through the domain ontology, has been a relevant step to support the next sense-making process. In fact, it is possible to formalize properties (e.g., vulnerability associated to assets) which exist in nature, thus reducing the complexity of the system description.

**Figure 6:**
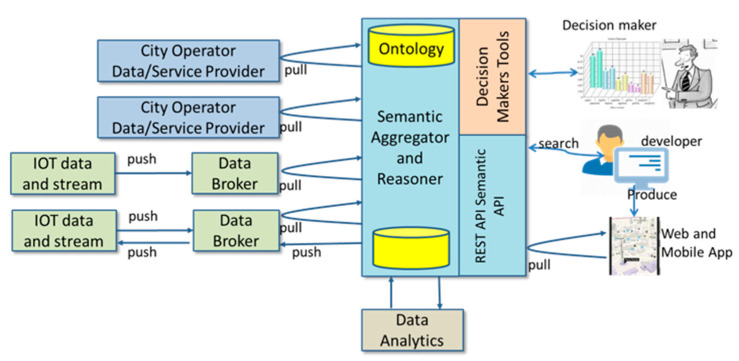
Data aggregation system.

The data aggregation system is illustrated in Figure 6, where the semantic aggregator and reasoner collect data from data sources, to fuse it in a unified and semantically interoperable model based on Km4City multi-domain ontology. Such a semantic aware data fusion approach reduces representation inconsistencies and overlaps of information coming from different structures, operators, and sources, [59]. The km4City multi-domain ontology has been adopted to model city entities and their logical and physical relationships enabling the inferential processes in the resulting knowledge base-KB (RDF Graph Database [57,58]). The KB can be used to create strategies for Data Quality improvement [60,61] as well as to set up new cross-domain services (e.g., early warning [62]). The obtained KB can be used to design new smart services (e.g., multimodal contextual routing) as well as support decision makers providing data driven integrated views of the city metabolism. Some existing approach fit this case as: CitySDK grounded on OASC (Open & Agile Smart Cities), Km4City [58,63], which is exploited by Sii-Mobility Smart City project and provides Smarty City API [64]; and SPUD [65], a commercial-based solution proposed by IBM.

These solutions require tools to map data to ontology to support the reconciliation as performed by DataLift in [66] and by Km4City in [58]. In both cases, vocabularies, algorithms, and dedicated languages have been used, as in SILK [65].

The creation of an ontology to support city resilience and impact assessment, expanding the pre-existing Km4City Multi-ontology, required a deep dive into the data sets available in the city, in order to identify the informative potential, establish the needed semantic relationships. The analysis of the above-mentioned data sets allowed us to create an integrated ontological model organized in eight main macro-classes as: Administration, Street-guide, Point of Interest, Service, Local Public Transport, Sensors, Temporal, Weather (see Appendix A).

#### 4.2.3. Ontology Extension for Dynamic Damage Analysis

The Km4City multi-ontology has been extended with new concepts dedicated to manage risk and resilience at city level. The objective was to design a semantic aware data model able to support real time risk and resilience computing and to extract information related to the level of damage occurring in an urban area. The impact is computed by considering different kinds of city asset exposed (services, assets, people, etc.) and their operational status at the time of the event. In fact, the value of a service (e.g., school) that is closed at the time should be considered reduced and the resulting damage quantification should vary accordingly. The scope is to support prompt and well-informed decisions to cope with the emergency. The damage is calculated with the product of vulnerability for the value of the exposed element in relation to a critical event with a certain intensity/magnitude. Therefore, the damage calculation depends on:

- Asset vulnerability: depends on the type of asset, its location, its physical characteristics (materials, design), and so forth;
- Asset value: it depends on its characteristics and functions and it has an economic and/or social value according to the role played in the society;
- Magnitude of the event: the magnitude of the event is calculated through specific Observation collections.

Observation: this class models the observations (measurements) performed by a Sensor. To manage the damage analysis the following observations have been included in the ontology:

- PluviometricObservation⊑km4c:Observation models instances of rainfall observations received by the related sensors;
- Thermometry⊑km4c:Observation it models instances of ground temperature received by the related sensors;
- EarthquakeObservation⊑ km4c:Observation it models instances of seismic observations received by the related sensors;
- TrafficObservation⊑ km4c:Observation it models instances of traffic observations received by the related sensors;

Geometry: The geospatial ontology (http://www.opengeospatial.org/standards/geosparql) has been used for the geometry class to model areas and points. A geometry is associated with a sensor entity to define its position or to other physical elements of the city to describe paths (cycling paths, bus lines, etc.).

Asset: This class models the main elements considered in the city such as services, people, infrastructures, etc. An asset is a something in the city that can be exploited.

km4c:Service ⊑risk:Asset

AssetValue: it defines the value of the asset. It includes:

(a) PhysicAssetValue used to model economic values/importance for the population related to business/physical asset;
(b) ServiceAssetValue used to model economic values/importance for the population related to the type of service;
(c) SocialAssetValue to model the value and/or the social importance related to asset/service.

Vulnerability: this class is used to model the levels of vulnerability of an asset. Each level of vulnerability is characterized by the following features: geographical area, asset type; links to a type of indicators; and reference to a range of indicator values.

Indicator: this class models a sensor indicator and offers insight on what it is observed by that sensor.

The resulting extension of the Km4City ontology can be validated and exploited with a SPARQL query-based process. In fact, with the proposed extension, is possible to study simulated scenarios or perform real time monitoring by linking to each sensor one or more geographic regions. The sensor observations are used to calculate the damage the event has caused to assets within the target area. For example, the following SPARQL query allows you to estimate the impact of a 4.5 intensity seismic event in a certain area by identifying all the affected assets (managed with the Km4City: service class) and their values (managed with the risk extension: hasAssetValue).

SELECT * WHERE {
?v a risk:SeismicVulnerability;
risk:forAssetType “Service”;
risk:fromMinIntensity ?min;
risk:toMaxIntensity ?max;
gis:hasGeometry/gis:asWKT ?wkt.
FILTER(?min ≤ 4.5 && 4.5 ≤ ?max)
?s a km4c:Service;
geo:lat ?lt; geo:long ?ln.
?s risk:hasAssetValue ?avalue.
FILTER(st_intersects(st_point(?ln,?lt),?wkt))
}

Such queries can be pre-configured and executed on demand for what-if analysis or as a close to time processes in case of emergency management. The approach exploits the data integration and the semantic-aware interconnections among the entities modeled in the knowledge base.

#### 4.2.4. Data Transformation Multi-Steps

The data aggregation sub-layer elaborates data coming from the data acquisition sub-layer and makes a set of transformations steps than can be resumed in the following actions:


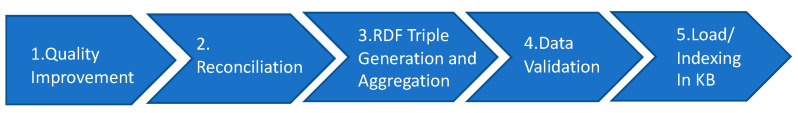


1. Quality Improvement, QI: since the ingested datasets may present different errors and inaccuracies. This is the reason why it is crucial to enhance the data quality, so as to produce reliable and useful information/knowledge for their composition and exploitation.

2. Reconciliation: in this step the lack of coherence and relations among entities referring to the same concept but coming from heterogenous datasets and data providers, is addressed. In fact, entities can present mismatch in semantics on names of the elements, dates, GPS coordinates, ZIP code, e-mails, telephone numbers, area codes, etc. For instance, the methodology adopted for the service consists in trying to link each service (Km4City: Service) to the street and civic number; the subsequent attempt to carry out the reconciliation at street-level, and at the street segment level.

3. RDF Triple generation and Aggregation: this step generates RDF triples for every static and slow-dynamic datasets, while the real time data are collected in a NoSQL database. The triples are stored in the RESOLUTE semantic data storage and are based on Km4City multi-domain ontology (http://www.disit.org/km4city/schema) whose aim is to semantically fuse all the information coming from the data acquisition sub-layer in an RDF data store.

4. Data validation: in this step verification and validation techniques to check correctness and consistency of the data and their relationships have been applied through a SPARQL query-based validation of RDF triples has been performed. Such a validation allows the verification of the fundamental constraints and the correct execution of multi-reasoning (geo spatial, temporal, and semantic).

5. Dataset load/indexing into the knowledge base, it loads the RDF triples on the knowledge base.

At the end of this processes, the enriched and fused data are made available to the upper tiers through specific RESTfull APIs, separating the activity of data management from that of analysis.

### 4.3. Tier II Information—U-Bmd Sense Making

This tier is devoted to transform data into information through sense making processes. To this end, data analyses have been applied such as: (1) human behavior analysis; (2) social media analysis; (3) predictions, etc. Each of these methodologies, described in detail in this section, represents a signal to be used in preparing and learning as well as responding and recovering phases.

#### 4.3.1. Wi-Fi-Based Human Behavior Analysis Module

**Figure 7:**
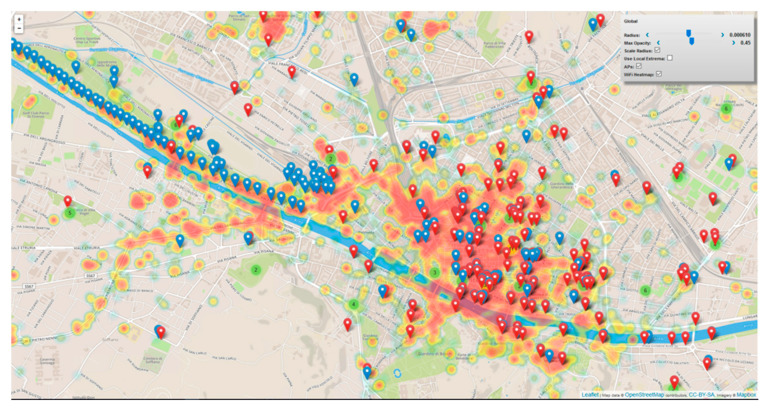
Florence heat-map. This heatmap put in spatial relation city users’ most patronized places with the position of the 1500 Wi-Fi APs of the whole network (using a color gradient scale to discriminate between different densities of measures).

According to [67,68], human mobility is proven to be highly predictable, since 85% of the time a mobile user stays in his/her top five favorite locations. However, under emergency, human behavior may be highly unpredictable, because of fear, wrong heuristics, lack of information, altered risk perception, etc. In this scenario, public Wi-Fi networks can be used to track city users’ behavior in space and time (see Figure 7). In this respect, Florence Smart city has implemented a free Wi-Fi network (Florence Wi-Fi) deploying more than 1500 Aps on the territory. Some of these Aps (about 365) have been instrumented for the user connection’s tracking. The data of device connections can be put in relation with a movement behavior. For the analysis, we referred to data collected in a period from May 2016 to May 2018 with about 400 K single events per day including connection and disconnection. The analysis found that the 60% of such connected users are tourists that use the free city Wi-Fi for less than 24 h. In 6 months, about 1.15 M distinct users have been detected, which means about 2.3 M of distinct users per year in a city having about 14 M of new arrivals per year and 350 K inhabitants. According to these figures we could infer that we tracked about 16% of people flows.

**Figure 8:**
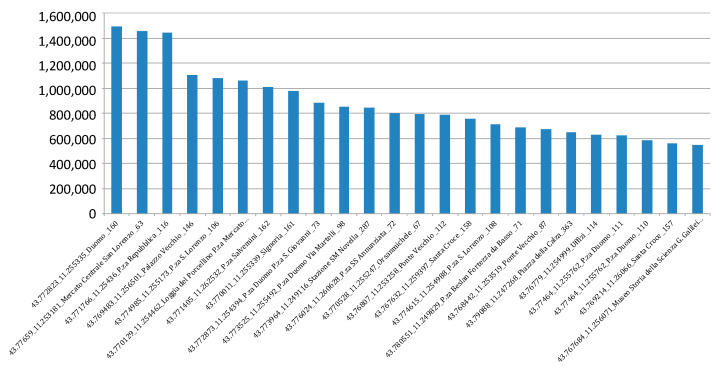
Distribution of hottest places in Florence (truncated series). The chart shows the cumulated number of Wi-Fi accesses in last 180 days (*y*-axis) associated to a lat/long of the access points distributed in the city (*x*-axis).

This data may represent a valuable source of information and knowledge. For instance, has been identified the “hottest places” (in terms of events on the APs) as reported in Figure 8. Places are represented with GPS (Lat/Log) data. This result provides us insight on how the people use the city in a certain time window. Knowing where the people are increase the possibility to respond to a critical event in an efficient way.

Every day, the WIFI network detects about 34 K distinct users of which 10% are new users. In Figure 8, is depicted the distribution of the so-called hottest places in Florence (cutting the list of the first 12). The city usage along the daily 24 h have been recorded and different trends have been clustered into 12 distinct clusters revealing strong differences among them. For example, there are zones where there are picks in the morning (arrival) and in the late afternoon (departure). In other areas (e.g., gardens), the presence density increases in the mid-morning and mid-afternoon while for lunch there is minimum. Thus, there are APs that experience a relevant workload during the early mornings and late afternoon only, others are stressed mainly on late evenings (time), others during the on-weekend days, and so forth.

**Figure 9:**
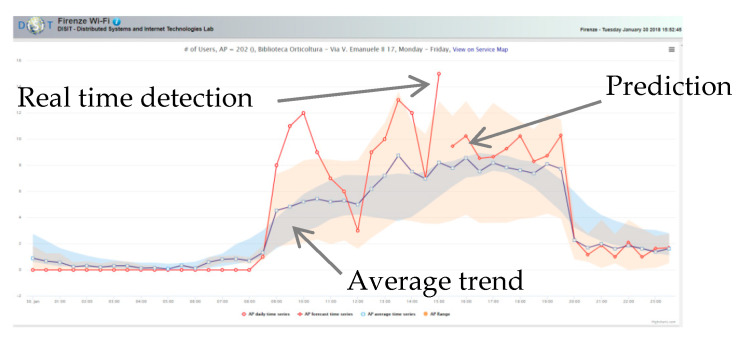
An example of trend related to a certain AP along the daily 24 h (blue line), the current detection (red line) and the subsequent prediction (red line after the gap).

In particular, the trend depicted in Figure 9 (blue line) has been extracted from 56 million of data records and estimated by computing the averaged value per time slot of the AP every working day. This trend has been compared with the one detected in real time (red line) in a specific day.

An anomaly is detected in case of relevant deviations from the average. It is worth to remark that it is not possible to infer the cause of such a deviation using only this source of data. To this end, a multi-source approach is necessary.

In particular, in emergency conditions, is it crucial to predict the people distribution in the city and infer patterns in city user’s behavior in order to have a reliable baseline for comparison (expected vs. actual). The approach is based on data mining techniques which cluster APs on the basis of their normalized temporal pattern. This process groups city zones according to their usage. Clustering the zones on the base of the APs’ data can be of great help in understanding if there are zones that exhibit similar exploitation or flow patterns.

In particular, the averaged trend along the 24 h of a day has been calculated for each AP of the 345 instruments for the tracking and for each day of the week. Then a scale factor and the normalized averaged pattern (from 0 to 1) have been computed. We focused on 7 days divided in 48-time slots per day (30 min). The first AP patterns emerged from the analysis has revealed a marked difference between the working days and the weekend. Thus, we decided to cluster such time series by considering three distinct groups: Saturdays (Sa), Sundays (Su), and the working days (W). From a statistical perspective, the temporal pattern for each AP has an average and an interval confidence for each time slot (as reported in Table 3).

At this point, a clustering process has been applied to identify similarities in time series, as it occurs in the dynamic time warping [69], and by using different clustering algorithms such as K-means clustering algorithm [70], hierarchical clustering [71], density-based clustering or subspace clustering [72], and metrics to evaluate both a better ranked clustering algorithm and proper number of clusters.

Another valuable analysis to understand city usage and human behavior that can be performed with U-BMD data is represented by the origin–destination matrix generation. The OD matrix representing flows among the areas of the city can be defined as

$$\mathrm{ODn},n\;=\;\left(\begin{array}{ccc} a_{1,1} & \cdots & a_{1,n}\\ \vdots & \ddots & \vdots \\ a_{n,1} & \cdots & a_{n,n} \end{array}\right)$$

where: a_i,j_ represents the total number of people flowing from a_i_ to a_j_ and is defined as

$$a_{i,j}\;=\;\sum_{t\in T}n_{t}\left(i,j\right)$$

*T* is the set of unique people flows, *n_t_*(*i, j*) is the number of traffic counts from a_i_ to a_j_ for the trace *t*.

**Figure 10:**
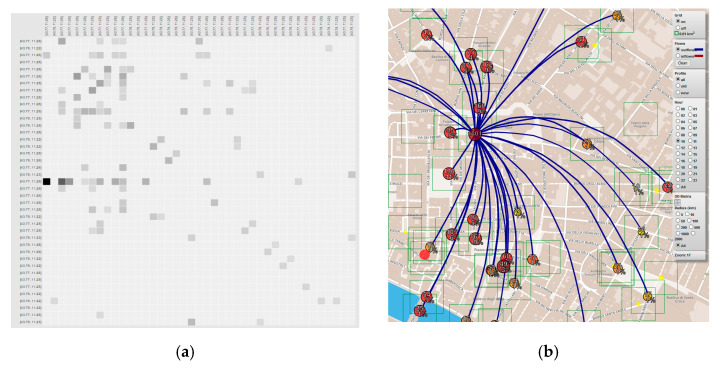
OD matrix for Florence downtown. In the OD matrix can be provided as: (**a**) classical view; (**b**) advanced interactive view where is possible to visualize inflow, outflow, time slot selection, user kind, and so forth.

In case the OD matrix calculated exploiting APs data, what can be actually evaluated is the intensity of the flows while the OD vertex are considered fixed since they are represented by the position of the APs. Moreover, the OD matrices are typically quite widespread, as you can see in Figure 10a, where the OD matrix for Florence is reported. It is evident that the city is used in a different way in different areas (see Figure 10b). In fact, AP areas present different kinds of trend in terms of people density along the daily 24 h and along different days of the week [68,72].

The analysis can be carried out for each time slot of the day or full day, for incoming and outgoing flows, and at different levels of resolution (zoom). The information generated by the OD matrix can be used to reduce uncertainty about people behavior while moving in the city. Therefore, knowing that a critical event occurs in an area that at that moment is likely to be affected by a flow of movement of people, will allow a more effective response in terms of resources employed (all those really necessary to manage the volume of people) and timeliness.

#### 4.3.2. Social Media Analysis: Twitter Vigilance Module

The datasets derived from social media are basically obtained from Twitter. Data are collected using the RESTFull APIs provided by the social network itself. Twitter generates a huge stream of information, and thus it is crucial to have a filtering strategy in advance in order to collect only the relevant information only and reduce the noise.

**Figure 11:**
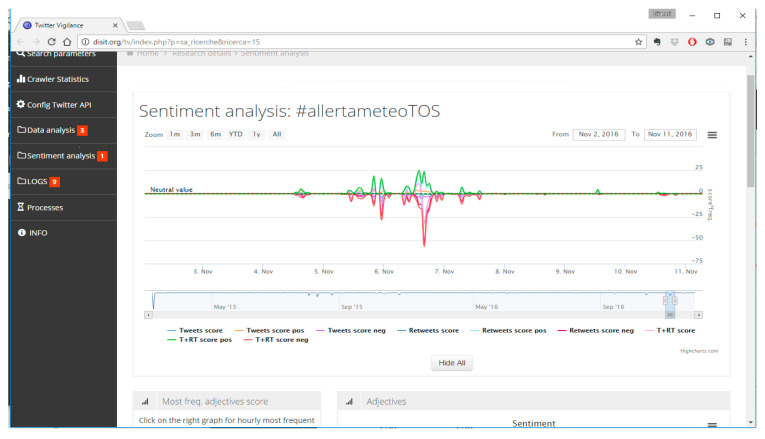
Sentiment analysis signal processing.

To do so the Twitter Vigilance (http://www.disit.org/tv/) tool (TV), developed in DISIT lab and used as a RESOLUTE project, has been used. The tool is able to collect, process, and analyze twitter streams by both defining filters (channels) and applying techniques of Natural Language Processing (NLP). The scope is to detect relevant variations in the defined channel in terms of numbers of tweets, the sentiment, and so forth. For example, the Figure 11 represent the interface of TV where a significant variation (negative) on the sentiment feature in the defined channel “Meteo” (Weather) has been detected.

**Figure 12:**
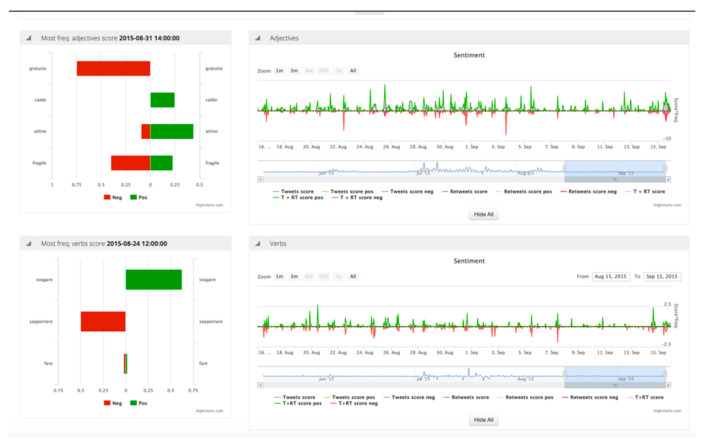
Sentiment analysis terms exploration.

The TV analyses the channel looking for nouns, verbs, adjectives, and deciding the acceptance level (negative/positive) of such terms. The Figure 12 represent another example of to a channel dedicated to monitor twitter related to heavy raining. In this case, the crowdsourcing based on Twitter allowed the detection of an issue in the city caused by the rain before the official authorities did. This means that, if properly managed (e.g., detection of strong increment of tweets and retweets), Twitter can be considered a social sensor for early warning mechanism.

**Figure 13:**
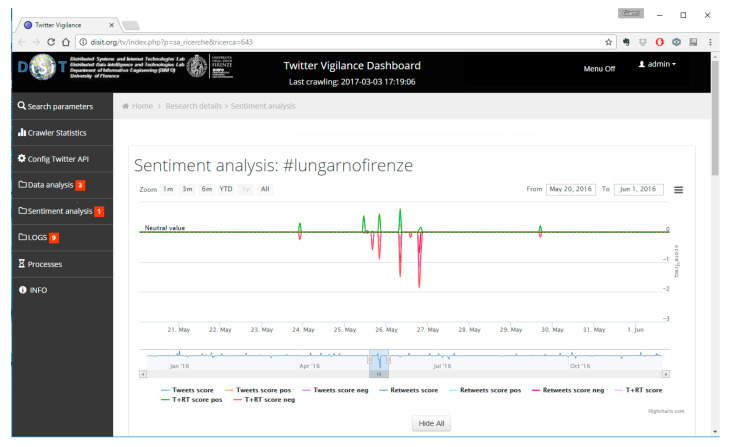
Sentiment analysis on Arno River situation.

However, crowdsourced data must be treated carefully. In fact, the assessment of the reliability of the information provided is still a challenge. In this respect, some criteria can be applied as the actual presence of users on the ground acting as a sensor (e.g., Twitter georeferenced). On the other hand, the absence of people when an event occurs, does not make the Twitter a reliable source for early warning since no one is present to report that is happening. This is exactly what happened during the Arno river embankment collapse in Florence caused by a water pipe disruption (see Figure 13). The event started at 6:15 a.m. on 25/05/2016 but no variation had been detected on TV. According to the Figure 13, the first relevant variation had been detected only in the afternoon of the same day. Another increment had been detected hours later, and in the days after, with a number of retweets because of the popularity of the place.

**Figure 14:**
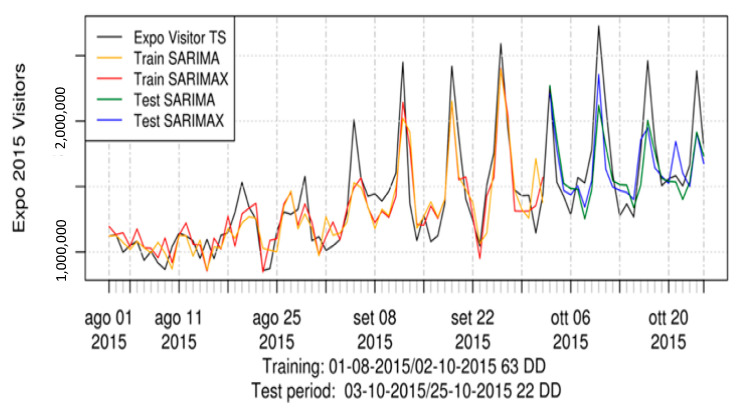
Twitter Vigilance predictive models for people attendance at EXPO 2015. The picture shows the comparison results among different models built on the collected Twitter Vigilance data and the real number of registered visitors.

Once the conditions for data reliability are defined, predictive models on Twitter dataset can be applied. Recent case studies and applications have been developed at DISIT Lab [73,74], using high-level and low-level metrics which were defined and monitored thanks to the Twitter Vigilance analysis tool. The latter has turned out to offer very good predictive capabilities in several different domains, for instance, predicting people’s attendance to large public events (such as EXPO 2015, see Figure 14), as well as predicting the audience of popular TV programs and shows.

According to this analysis and other related experiences [74], it is noteworthy that using social media data represents a powerful tool requiring a deep understanding of its dynamics, its capacity of meaning, and the related method on how to extract such meanings.

**Figure 15:**
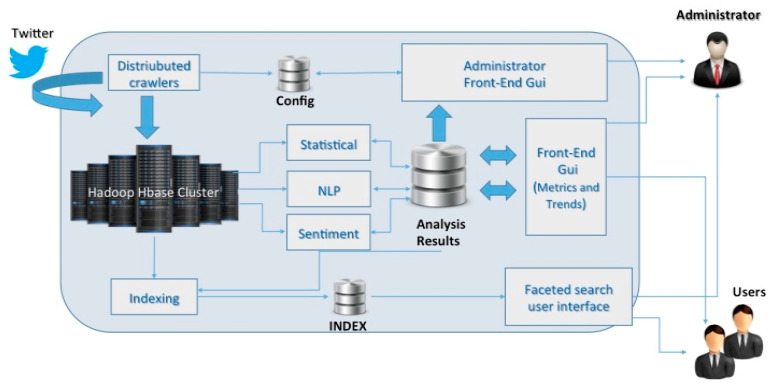
Twitter Vigilance architecture.

Since the social media data have the characteristics of the Big Data (4V), a dedicated architecture based on distributed crawlers executed on a cluster of N nodes has been implemented for Twitter Vigilance (see Figure 15). The data acquisition is based on the concept of Twitter Vigilance Channel (TVC) that acts as a filter. The TVC consists in a set of searching queries based on the combination of some parameters as keywords, hashtags, user’s IDs, citations, and so forth. The collected tweets are elaborated through back-office processes based on statistical analysis, NLP, and sentiment analysis, as well as general data indexing. TV users can define and monitor the high-level metrics and set up custom rules to produce alerts and other customized actions. Every metric can be calculated at different time frame (daily, hourly, and real-time) according to the user needs. The metrics calculated are stored in a dedicated SQL database and made accessible to the front-end user interface. The TV interface allows the customizing of the search query, implements dashboards, provides reports and file export features (e.g., to CSV format) for visual analytics, temporal trends and time series visualizations, data results navigation, Twitter user’s statistics and analysis.

### 4.4. Tier III—Knowledge-Driven Support System

The last tier is represented by the decisional layer. It has been supported by the implementation of the Resilience DS tool, an evidence driven decision support system (EDDSS) that exploit the information and knowledge generated by the Tier II to quantify.

**Figure 16:**
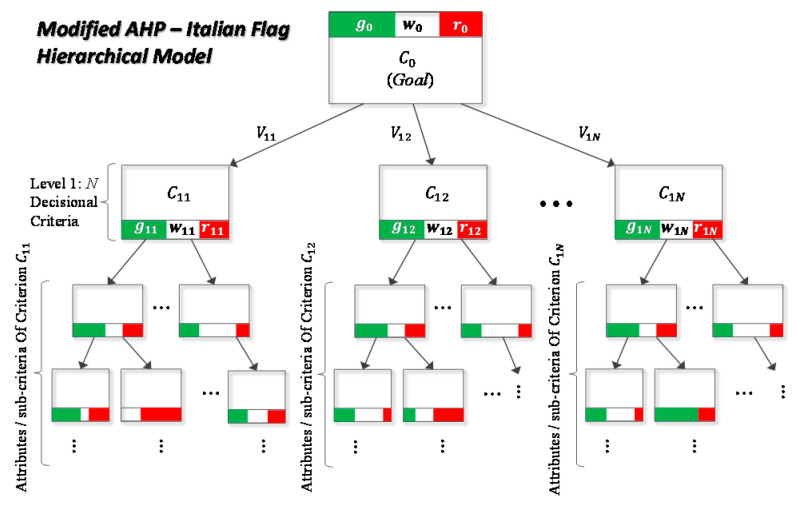
AHP–IF hierarchical model. General schema of the modified AHP hierarchical model integrated with IF representation.

This tool is built on top of the SmartDS tool [74], an Evidence Support Logic (ESL) based tool that uses the Italian Flag (IF) Representation [75] as a three-values logic to measure uncertainties. A general schema for the modified AHP (analytic hierarchic process) model, including the IF representation, is shown in Figure 16.

#### Resilience DS

The Resilience DS is a collaborative tool inspired by System Thinking [76] view that integrates the FRAM methods in a decision support system (DSS) fueled with data and experts’ judgments. The Resilience DS tool moves the FRAM modeling approach towards its computability and quantification, by introducing several formal checks to assess the completeness, consistency, and complexity of the model and to connected it with the data make available by the Tier 2 [77].

SmartDS operates with two entities: models and instances [74,76]. A model identifies a set of decisional criteria modeled as a hierarchical decision tree and having the root standing for the goal to be achieved (see Figure 16). The nodes belonging to the first level of the tree represent the decisional criteria which have been defined to achieve the goal. Lower-level nodes can describe sub-criteria, as well as properties of corresponding upper level criteria: the factors that contribute to take the decision.

In the next step, the weights related to each criterion of the decisional tree should be estimated. This is performed by using the evaluation matrix, whose single elements are obtained by pairwise comparisons of the decisional criteria. Considering a generic level $\tilde{l}$ of the hierarchy, composed of *N* criteria $C_{\tilde{l}1},\ldots ,\;C_{\tilde{l}N}$, the pairwise comparison matrix is defined as

$$P_{\tilde{l}}=\left[\begin{array}{ccc} p_{11}^{\tilde{l}} & \cdots & p_{1N}^{\tilde{l}}\\ \vdots & \ddots & \vdots \\ p_{N1}^{\tilde{l}} & \cdots & p_{NN}^{\tilde{l}} \end{array}\right],$$

where elements $p_{ij}^{\tilde{l}}$ (*i*, *j* = 1, …, *N*) are the Saaty’s scale values representing the comparisons among criteria. The comparison matrix *P* has the property according to which its symmetrical elements stand in a reciprocal relationship (in agreement with the Saaty’s rating scale)

$$p_{ij}^{\tilde{l}}=\;\frac{1}{p_{ji}^{\tilde{l}}},\;1\leq i,j\leq N$$

Subsequently, a normalization by column is made over P, thus obtaining the $P_{\tilde{l}}\;$matrix. Under the assumption of having *N* nodes at level $\tilde{l}$, the $\hat{P}\;$matrix is defined as

$${\hat{P}}_{\tilde{l}}=\left[\begin{array}{ccc} {\hat{p}}_{11}^{\tilde{l}} & \cdots & {\hat{p}}_{1N}^{\tilde{l}}\\ \vdots & \ddots & \vdots \\ {\hat{p}}_{N1}^{\tilde{l}} & \cdots & {\hat{p}}_{NN}^{\tilde{l}} \end{array}\right]=\left[\begin{array}{ccc} \frac{p_{11}^{\tilde{l}}}{\sigma_{1}} & \cdots & \frac{p_{1N}^{\tilde{l}}}{\sigma_{N}}\\ \vdots & \ddots & \vdots \\ \frac{p_{N1}^{\tilde{l}}}{\sigma_{1}} & \cdots & \frac{p_{NN}^{\tilde{l}}}{\sigma_{N}} \end{array}\right]$$

where

$$\sigma_{1}=\overset{N}{\underset{k=1}{\sum }}p_{k1}^{\tilde{l}},\ldots ,\;\sigma_{N}=\overset{N}{\underset{k=1}{\sum }}p_{kN}^{\tilde{l}}$$

Priority weighs $V_{\tilde{l}1},\;\ldots ,\;V_{\tilde{l}N}\;$are finally obtained by computing the arithmetic mean over the rows of the normalized matrix ${\hat{P}}_{\tilde{l}}$.

The next step is the assignment of weights to each node/factor. Weights are assigned on the basis of a mutual priority degree estimated among each pair of defined decisional criterion. Priority weights are computed, for each level, by comparing each pair of decisional criteria using the Saaty’s scale coefficients [74]. This rating scale assigns integer values from 1 to 9, according to the relative relevance between the compared elements. Such an operation leads to the creation of a pairwise comparison matrix for each level of the decision tree.

The IF representation has been integrated, since the belief that an event may occur, can be only partial. In this respect, a certain level of confidence should be considered and quantified. On such grounds, given a generic proposition or event E, the probability that it will happen(E), and the probability that it will not P(not(E)), the measure of uncertainty can be defined as: 1–P(E)–P(not(E)).

**Figure 17:**
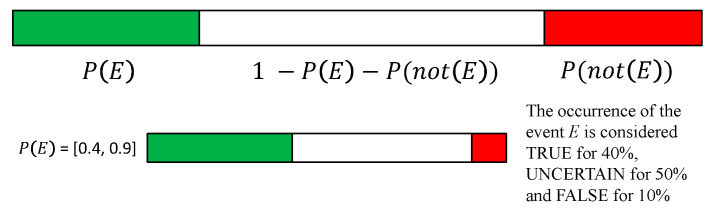
Three-value logic IF. This representation is for a generic proposition or event E with some examples explained.

Hence, IF is a simple graphical representation of the triple: [P(E), 1–P(E)–P(not(E)), P(not(E))]; where P(E) is represented with green, P(not(E) as red and, 1–P(E)–P(not(E)) is represented with white (see Figure 17).

Once the modified AHP-IF model is created, decision makers can create an instance of it. The instance identifies a hierarchical decision tree-based model fueled with data needed to compute the final decision. The data can be from different sources such as:

(1) Data from external sources obtained with HTTP API requests: the tool supports a semantic ware query to an external RDF semantic repository accessing to the SPARQL endpoint URLs and a query to a generic HTTP REST requests and calls to dedicated services/APIs. It is possible to combine up to two queries for each single node and the results are compared to threshold values defined by decision makers.
(2) Data from stakeholders’ opinions and feedbacks. In particular, opinions can be directly mapped to IF values: the value for the green color is obtained from the percentage of favorable opinions, the value for the white color is obtained from to the percentage of uncertainty opinions or answers not provided, and the value for the red color is derived by the percentage of opinions against that criterion/condition.

Once the instance is deployed, it continues to query data sources, process data and produce results adjusting the probability that an event occurs in close to real time. 

## 5. Results

According to the scenario-based design (SBD) technique [78], the flash flooding scenario is analyzed to demonstrate the enhancement in the system’s adaptive capacity obtained by using U-BMD when managing disaster resilience. The flash flooding is an extreme event which is going to be even more recurrent in areas that are not used to (and thus not prepared) for such extreme event. The main characteristics of the flash flooding scenario are:

(a) Impossibility to identify exactly the involved areas.
(b) Extreme intensity of rainfall
(c) Abrupt reduction of temperature and visibility
(d) Sudden overflowing of water on roads, underpasses, etc.
(e) Sudden traffic flow reduction

To cope with this situation the following decisions should be enabled to mitigate the impact and sustain a certain level of UTS service functionality:

- D1: send an appropriate rescue team in due time (if needed)
- D2: send appropriate street maintenance team in due time (if needed)
- D3: closure of the underpasses in the affected area
- D4: redirection of the incoming traffic towards alternative routes
- D5: provide safety recommendation to people in the affected area considering potential risks (e.g., high water in a specific area because of particular land shaping)
- D6: alert population about status of the event to orient their decisions (e.g., to discourage passage in the area)

To demonstrate the capability of the U-BMD to be exploited in disasters resilience management a subset of the FRAM-based model representing the UTS has been identified [2,50]. Indeed, during a critical event (absorption phase), all the UTS functions are involved in the absorption and recovery phases. However not all the functions are equally engaged. In the example we have identified the most relevant functions being at the forefront during an emergency.

M4—Monitor User-Generated Feedback: this function is devoted to monitor every kind of feedbacks generated by the service users to produce in real time, fundamental support to the deployment of operational adjustments. This function integrates in the in the context of operational monitoring, the assessment of user generated feedback to early detect issues and to take informed decision.

- Output: User-Generated Critical Event Detection

M2—Monitor Operations: this function is related to the deployment of a system operation performance assessment tools and practices integrating multi-stakeholders’ indicators with the overall service delivery needs. This turns out to be a key point to generate overall system performance understanding.

- Output: Critical Event Detection

R2—Coordinate Emergency Action: this function is dedicated to manage emergency during a critical event. The function is triggered by a M2 and/or M4 functions if a critical event is detected. The function has associated some decisions that should be taken. Such decisions produce outputs for other functions in the model.

- Output:
  - Rescue (D1,D2)
  - Operation Changes (OC) (D3,D4)
  - Advices (D5,D6)

A6—Manage Awareness and User Behavior: As providers of fundamental public services, critical infrastructures tend to be significantly exposed to individual and collective behaviors, in many cases not only the one by service end-users, but also by a wider audience. Recent ICT technological developments offer a great potential to enhance the audience interactions and the use of this potential for an increased effectiveness in managing and deploying operational adjustments to different relevant events and circumstances.

- Output: Early Warnings

**Figure 18:**
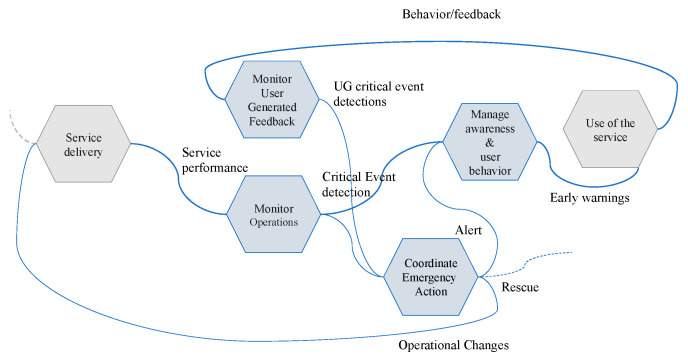
Sub-set of UTS FRAM model.

In Figure 18, such functions are represented with their interdependencies as extracted by the UTS general model presented in the ERMG [50]. In particular, monitor operation receives “service performance” as input from service delivery function which is not evaluated here (in grey). “Service performance” includes U-BMD from sensors like traffic, rainfall, conditions of underpasses, weather, etc. Similarly, the function “Monitor User-Generated Feedback” is received from the function “Use of the Service Feedbacks and Behaviors” as input. They are represented in terms of both presence and movement of people on the ground as detected with public Wi-Fi and social media like Twitter. The output of both functions is similar and is represented by the detection of a critical event: “Critical Event Detection” and “User Generated Critical Event Detection” respectively. The two outputs trigger the function “Coordinate Emergency Action” of which one of the outputs is “advice/command” to be processed by the “Manage Awareness and User Behavior” in order to forward an “Early Warning” towards the population. Then, to close the loop, the “Early Warning” is used as resource by people to orient their local decisions which are detected by the “Monitor User-Generated Feedback” function.

Decisional support for the Coordinate Emergency function can be provided through the SmartDS tool, in order to automate some decisional process relying on data analysis (for instance, data from sensors, users etc.). For example: (1) provided that the whole scenario is a complex model and assuming for each FRAM function several different inputs, resources, time, control, and preconditions entries (as described in Paragraph 3.2); (2) we hold that both Monitor Operations and Monitor User-Generated Feedback functions provide the following resources to analyze their inputs. Thus, according to the FRAM model the following inputs are taken into account by the SmartDS engine querying RESOLUTE Knowledge Base (KB) by using Smart City APIs and fueling the AHP-based models of the decisions to be taken in Coordinate Emergency function:

- Monitor Operations resources:
  - Traffic observation from sensors applying user defined thresholds on results to detect traffic flow trends, predictions, and reconstructions;
  - Underpasses water level observation: for underpass water level, applying user defined thresholds in order to detect if water exceeds the safe level in a given underpass;
  - Rainfall observation from pluviometry sensors at different times, applying user defined thresholds on results to detect if rain level exceeds a safe value;
  - Temperature observation from thermometric sensors at different times, applying user defined thresholds on results to detect if temperatures abruptly drop down;
  - Weather reports and predictions: related to temperature, dew point, humidity, etc.;
  - Pollution reports and predictions: related to environmental sensor data and reports;
- Monitor User Behavior and User Generated Feedback resources:
  - People density real time and predictions, established analyzing data coming from the city Wi-Fi sensors and resulting data analytics as previously described;
  - Prediction on parking lots collecting data analytic results and thresholding on the basis of their values.
  - Twitter Vigilance metrics: collecting volume, natural language processing and sentiment analysis metrics (as well as custom high-level metrics defined by users) about Florence weather related channels.

**Figure 19:**
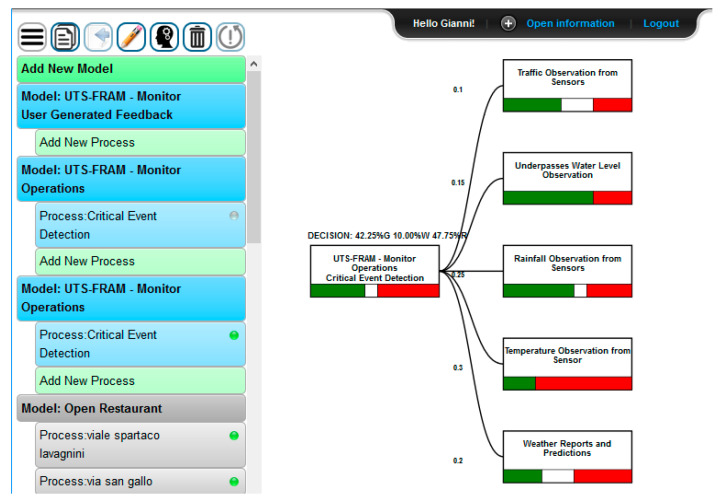
SmartDS decisional process implementation as to the Monitor Operations function (represented in the FRAM sub-set in Figure 16).

**Figure 20:**
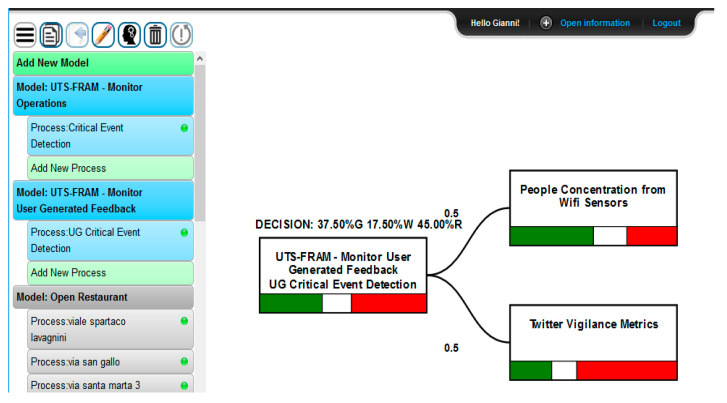
SmartDS decisional process implementation as to the Monitor User-Generated Feedback (represented in the FRAM sub-set in Figure 17). http://smartds.disit.org/dss/ also used in Snap4City https://www.snap4city.org.

The output of SmartDS computation affects the input of the Manage Awareness and User Behavior function, and therefore the Early Warning output signal. The implementation of the SmartDS computation for the above-described resources as to the Monitor Operations Resources and Monitor User-Generated Feedback is depicted in Figure 19 and Figure 20.

On such grounds, the level of activation of the early warning rely on the result of the weighted contribution provided by the Critical Event Detection and UG Critical Event Detection inputs plus other parameters (inputs, resources, preconditions, time, control) modeled in the Coordinate Emergency that, in turn, inputs the Manage Awareness and User Behavior decision tree. Because of the complexity of FRAM model (presence of loops, one output can be input for more than one function, etc.) and the needs of maintaining in the analysis a systemic perspective instead of a process-based perspective (where only specific paths are focused), it is not possible nor interesting to build a unique generic tree for the entire FRAM model. To this end, each function is computed separately and in parallel; and the results are propagated through the defined interdependencies with a step-based advancement. Hence decisions related to the R2 function are taken on the basis of the data driven event detection generated by the M2 and M4 functions, considered as a result of their parallel evaluation and the related outputs’ propagation in the model.

The improvement of the outputs of R2 decisions represents the real added value of the approach. For instance, the advice output forwarded by the R2 to the A6 function can be translated into tailored information to be provided to the city users at the right time and place via several kinds of channels: SMS, mobile apps [79], direct calls, message variable panels in relevant points of the city, tv news, radio news, social media, etc. The rescue action is now timely and appropriate since position and magnitude of the event are known in few seconds as well as the actual resource available to be allocated. The proposed approach brings several benefits in the resilience management. Such benefits have been assessed against the three main dimensions in which the output variability can occur such as time (T), precision (P), and confidence (C).

The results obtained in Table 4 shows the remarkable improvement obtained with the adoption of U-BMD in particular and smart technologies in general. The result is also comparable with the resilience improvement obtained with smart technologies and quantified in [2].

The introduction of U-BMD produces a relevant reduction of the variability experienced in the current condition, moving from suffering an interruption to managing a graceful degradation in service. This leads to better use of the resources available to manage the emergency and to a quick recovery. Finally, the possibility to save data represents a unique opportunity to build and share knowledge within and across the institutions involved in the UTS resilience management. In this sense, the adaptive capacity of the UTS results enhanced by the solution proposed.

## 6. Conclusions

The article aimed at presenting a full-stack approach to exploit U-BMD generated by the IoE in a smart city to enhance the UTS resilience management in the context of smart city. The challenge of managing U-BMD is meant to address the big data. In disaster resilience, timely decisions with reduced uncertainty are crucial for the application of adaptation strategies. To this end, a novel conceptualization for resilience suitable for its data driven operationalization and the related multi step methodology for its implementation that includes system understanding, information requirement definition, U-BMD collection and integration, U-BMD sense making process, and evidence-driven decision support system tools, have been defined. System understanding has been addressed adopting the FRAM approach, that is able to describe system functions and interdependencies. Once the model of UTS is defined, the information requirement elicitation from operators and decision makers has to be carried out. The system modeling and the information requirements allow the definition of criteria for U-BMD pre-selection. Such a U-BMD pre-assessment is crucial for cost reduction, especially management costs related to useless data collected only “just in case”. U-BMD have been managed through a three-tier platform including: (a) the U-BMD management layer, where heterogeneous datasets with different stream rates, volume and formats are collected and fused according to a specific ontology; (b) the U-BMD sense making for information extraction layer that includes a number of analytics applied on the single data streams such as: WIFI clustering applied on WIFI access data stream, Twitter Vigilance applied on twitter streams, Parking analysis applied on parking sensors data stream, etc.; (c) the knowledge-generation layer where ResilienceDS and SmartDS work together to model the UTS system according to FRAM methodology, so as to import the model into a three value logic decision tree tool able to link the models to data through APIs and queries. The reduction of uncertainty has been proven by tracking the evolution of white part of IF over time for most of the several formalized functions. From our analysis, this fact has been due to effect of formalization of the processes, which increased the awareness of decision makers and of experts, and velocity and precision for their assessment exploiting the big data tools. 

To conclude, this full stack solution aimed at supporting a timely and better-informed decision making in emergency as well as in daily operation within a complex system as UTS or a smart city itself, exploiting the U-BMD generated in a smart city. The operator can make data driven timely decisions in emergencies or in a planning phase (e.g., urban design [80], cost benefit analysis [81]) concerning the priorities for action, so as to safeguard the security of goods and people involved therein [82], while simultaneously studying which are the areas having a potential greater risk, as to the occurrence of an event, in order to implement preventive policies. However, it is worth to remark that the quality of the outcome provided by the system is intrinsically related to the quality of the data managed [60,61,83]. The future evolution of such system can be represented by the Digital Twin. The possibility to create a leaving virtual representation of UTS connected to real data, is at hand. The creation of a simulation models that continuously update and change as their physical counterparts’ change will allow a better evaluation of the decision space for resilience and a deeper understanding of the effect of such decisions.

## Figures and Tables

**Table 1:** FRAM-based UTS functions.

Anticipate	Monitor	Respond	Learn
A1—Manage financial affairs	M1—Monitor safety and security	R1—Restore/repair operation	L1—Provide adaptation and improvement insights
A2—Develop Strategic plan	M2—Monitor Operation	R2—Coordinate emergency action	L2—Collect event info
A3—Perform risk assessment	M3—Monitor Resource availability	-	-
A4—Training Staff	M4—Monitor user generated feedback	-	-
A5—Coordinate service delivery	-	-	-
A6—Manage awareness and user behavior	-	-	-
A7—Develop/update procedures	-	-	-
A8—Manage human resources	-	-	-
A9—Manage ICT resources	-	-	-
A10—Maintain physical/cyber infrastructure	-	-	-

**Table 2:** Enabled decisions–functions mapping.

Enabled Decisions	Anticipate	Respond	Monitor	Learn
When and if resource availability should be improved (e.g., operators, volunteers, funds, means)	A1, A2, A3	-	M3	-
Which kind and how many units must be dispatched during a critical event—better situational awareness.	A8,	all	M1, M3, M4	L2
When and where population should be evacuated to a safer area (respond);	-	R2	M4	-
Delivering timely and correct information to the public, etc. (respond, anticipate, learn);	A9	R2	M4	-
If/when suspending or redirecting public/private-transport-services (anticipate, respond);	A5	R1	M1, M2, M4	-
How much and when investing in infrastructure maintenance/improvement (anticipate, learn);	A1, A2, A3,	-	M1, M2, M3	-
Training population and enhance their awareness	A4, A6	-	-	L1

**Table 3:** Standard Deviation and Population for AP Clusters (W: Working days, Sa: Saturday, Su: Sunday).

Cluster Id	Avg. Std. Dev.	W	Sa	Su
1	0.2379	172	23	24
2	0.0849	23	43	43
3	0.0882	8	42	34
4	0.1820	3	30	26
5	0.1059	20	15	14
6	0.0822	38	15	8
7	0.1311	9	57	34
8	0.1374	2	23	55
9	0.1226	4	32	38
10	0.1460	52	12	3
11	0.2487	11	13	21
12	0.1617	1	28	31

**Table 4:** Evaluation.

Output	Variability without U-BMD			Variability with U-BMD		
	Time	Precision	Confidence	Time	Precision	Confidence
Critical Event Detection	Too late (>30 min)	Imprecise (based on operator reporting)	Mid	In time (s/RT)	Precise	High
User Generated Critical Event Detection	Not at all/ Too late (>30 min)	Imprecise (based on few reports of citizens)	Low	In time (s/RT)	Acceptable/ Precise	Mid/ High
Alert	Not at all/ Too late	Imprecise	Low/ Mid	In time (s/min)	Acceptable/ Precise	High
Rescue	Too Late/ In time	Acceptable/Imprecise	Mid	In time	Precise	High
Operation Changes	Not at all/ Too late	Acceptable/ Imprecise	Mid	In time (s)	Precise	High
Impact	- UTS service disruption - Slow recovery - Human experience-based learning for adaptation			- UTS graceful degradation for the service survival - Quick recovery - Evidence-driven learning for adaptation		

## Data Availability

Data available on request due to safety and security concerns.

## Appendix A

**KM4City Macro Classes**

**Administration**: it includes classes related to the structures of any public administration. It includes the class Resolution, which represents ordinances and resolutions issued by each administration and meant for instance to change any traffic stream. As an example, a Description Logic based axiom is here reported:

Municipality ⊑PublicAdministration ⊓(∀isPartOfProvince.Province)…

Province ⊑PublicAdministration ⊓(∀hasMunicipality.Municipality)⊓(∀isPartOfRegion.Region)…

**Street-guide**: it includes entities such as Road, Node, RoadElement, AdministrativeRoad, permitted maneuvers and related access rules to the limited traffic zones, and so forth. Because of the complexity of the entity, the OTN vocabulary [84,85], has been used to model traffic which can be considered a direct encoding of GDF (Geographic Data Files) in OWL (https://www.w3.org/OWL/). Some DL based axioms are described here below:

Road ⊑otn:Road ⊓

(∀containsElement.km4c:Element)⊓

(∀isInMunicipality.km4c:Municipality)…

RoadElement ⊑otn:Road_Element⊓

otn:Edge⊓

(=1 startsAtNode. Node)⊓

(=1 endsAtNode. Node)

**Point of Interest** (PoI): includes place as well all services and activities. The classification of individual services and activities is built on main and secondary categories planned at regional level. In addition, this macro segment of the ontology may benefit from reusing the Good Relation model of commercial offers. For example, a DL axiom is:

Hotel ⊑Accommodation⊑Service⊑otn:Service

**Service**: it is a classification of services in KM4City, from banks to schools classified in about 20 classes and more than 530 subclasses.

**Local Public Transport**: includes the data related to LPT (Local Public Transport) companies, scheduled times (modeled with General Transit Feed Specification-GTFS), the landrail logical and physical graphs (SHP file), and real time data related to the passages at stops. The scheduled timelines are modeled according to. For example:

BusStop ⊑gtfs:Stop

BusStop ⊑TrasferServicesAndRenting

**Sensors**: it models the sensors installed in the city and the related data. A critical sensor data stream is that related to the traffic. For example:

TrafficObservation ⊑Observation ⊓

(∀measuredBySensor.SensorSite)⊓

(∀instantObserv.time:Instant)…

**Temporal**: macro class which updates concepts related to time (time intervals and instants) within the ontology, thus enabling to associate a timeline with the recorded events and allowing forecasts. It may benefit from time ontologies such as OWL-Time, [85].

**Weather**: it collects all weather reports and prediction generated by collecting heterogeneous meteorological data ingested by different kinds of sensor (temperature, pluviometry, humidity, wind intensity and direction, snow, etc.).
